# Family Taints

**Published:** 1879-09

**Authors:** Samuel Peters

**Affiliations:** Cohoes, New York.


### ARTICLE IV.

### *Family Taints.*

BY SAMUEL PETERS, M. D., OF COHOES, NEW YORK.

The fact has often enough come to the observation of physicians, that some families of children have a low or weak vitality. Such families frequently, and I think as a rule, contain several members, even more than the average, so that we might term them large families; but they die, some with one disease some with another. Prominent among such fatal diseases we may name tubercular meningitis, scarlet fever, diphtheria, cholera infantum, etc., although almost any disease is likely to overpower the tender body and prove fatal. Thus we often see several little graves in a row, one after another having fallen a prey to some affection that others would have readily overcome.

What the peculiar constitutional character of such a family is, may in some cases be difficult to understand. We find, as a rule, the children are fair, intellectual, promising, even sometimes stout and rosy-cheeked, light skin and hair, yet possessing a certain *taint* which readily proves itself in unmistakable evidences of low grade of vitality, and for which, I believe, we have no name that will serve for all cases.

It may be remarked that the objective signs just named are not always present (Miller, Roberts.) Some have dark skin and dull intellect.

I have observed that the parents may, and often do, appear to be quite healthy, with no indications, even to a physician's eye, that they must foster such a progeny. And what is more strange, they may not be in the line of any special hereditary disease, yet nearly every child seems to be doomed, sooner or later.

I have further observed that if one of the children was saved it was much more frequently the first—this to grow up perfect, or more or less feeble—the succeeding ones falling, one after another. Sometimes one, two or three survive, fortunately, but generally many more die than live; only a small part living to grow up.

It is certainly disheartening to the physician to observe such sad waste; families decimated, children dying in spite of all the known means that are at his command; and, indeed, he is often blamed for his ill success, when he has exercised a skill and judgment than which no better could have been found. Such sad mysteries, if the expression be allowed, sometimes confront us, and will continue to do so until we learn more of the great laws of reproduction.

However, some cases are clearly enough traced to their true cause; one or both parents exhibiting distinct evidences of loitering disease; or, though healthy themselves, yet falling directly in the line of hereditary disease. Or again, there may be a peculiar kind of exhaustion, which will be noticed hereafter. When such causes can be found our embarrassment is, of course, at an end. We say to the parents, "Your children are *tender;* they cannot endure as much as other children; they are like a vine growing in the cellar, easily snapped, and if you bear others, they also will be liable to fall as easily." It is, indeed, well that such a satisfactory explanation greatly mitigates their distress of mind.

But such families are occasionally found where no evident cause can be assigned. The parents are able to boast of uninterrupted health, and of entire freedom from hereditary disease; their ancestors have lived to a ripe old age, perhaps, and where the fault or failure exists cannot be told with any degree of satisfaction.

Among the family faults, or "taints," as they have been named, rather carelessly, perhaps, are scrofula, rheumatism, syphilis, general anæmia, and what we now term sexual exhaustion. I shall not take time to discuss these at any great length, only to make a few remarks on the first and last named.

While all these faults have more or less to do with the mortality among infants and children, it is undoubtedly the fact that scrofula is the most potent factor in the relation of cause, vitiating, as it does, the human system in such a manner that it is rendered incapable of withstanding disease to the extent that it otherwise would. In a word, scrofulous children are tender, delicate, frail; not in external appearance, perhaps, as already remarked, for they may be rosy-cheeked, even plump; "very healthy," as the parents will often say, but so in fact, as proved by the readiness with which they are swept away.

Now, all our observations, I believe, tend to confirm the opinion expressed by Rindfleisch (Ziemssen's Cyclopedia, vol. v.,) which is based upon the researches of Virchow, regarding inflammatory exudations in scrofulous persons. I cannot do better than to quote from page 636 of the volume named: "Virchow first called attention to the predominant cellular character of the scrofulous exudation; to its hyperplastic nature, and to the low vitality of the cells which compose it. For my own part, I would add that fresh scrofulous exudations contain relatively *large* cells, with glistening protoplasm, and a nucleus in the act of segmenting, or containing a double nucleus. I have received the impression that the emigrated white blood globules, which in normal individuals pass from the blood vessels of the inflamed tract to some adjoining surface, or to the lymphatics or lymphatic glands, or become collected into abscesses, have a tendency, in scrofulous persons, to grow larger on their way through the connective tissue. They swell up by the intussusception of albuminous substances, and in this very swelling die and slowly degenerate. The consequences of this peculiar anomaly of vegetation are felt in all the inflammations of scrofulous persons; less in the superficial catarrhs of the skin and mucous membranes than in the deeper parenchymatous inflammations of the glands and viscera."

If this be true, that all inflammatory processes in the scrofulous are almost sure to pursue such a course, we may readily indicate to our own minds the cases in which lie the greatest danger; for whatever disease the scrofulous system encounters that has as a part and parcel any inflammatory process, of whatever surface or tissue, that disease will result, in a greater or less degree, in the complications and dangers just described. Hence, we have long ago learned to dread, in the scrofulous, the diseases to which children are especially liable which have an inflammatory or even a hyperæmic character. The same explanation, I have no doubt, can be given of the serious results which sometimes follow vaccinations in these subjects, even with the purest virus. No amount of care can always avert such unpleasant consequences. All are aware of the great difference in the results which follow ordinary injuries, as, for instance, of the joints, or other parts, the difference being determined by this deep-rooted all-pervading constitutional taint.

But another cause may undoubtedly be assigned for the low vitality in children of certain families; a cause which is now and then simply mentioned, but not really dwelt upon and distinctively urged, yet I believe a very prolific one in inducing the bodily condition under consideration. I refer to sexual exhaustion, commonly induced, as is well known, by the frequency or rapidity of conceptions; also by the ruinous practice of imperfect or incomplete coitus, and perhaps by repeated abortions. I cannot but think that sexual exhaustion as a cause of low vitality is not receiving its due share of attention; that the public is not properly enlightened, through the profession; that we are perhaps criminally negligent in failing to discharge so vitally important a duty, while it is carrying off, probably, as many as all the other causes we have named, except, possibly, scrofula, and even this, with all its evils, may frequently be engendered by it.

Sufficient, however, has been said on these points, as my principal object in this paper was to speak more especially upon the physician's part in the management of such families, by moral, hygienic, and medicinal treatment. That we may do much to fortify such constitutions against what may, and, indeed, must be, encountered, is admitted by all. Not that we can in all cases entirely eradicate the cachexia, but that we may so far neutralize its influence or power that the system will not so easily succumb to the influence of disease. In other words, that we may raise the grade of vitality, and perhaps, even, in some cases, place it on a level with the strongest.

The hygienic management of such families is all important, and pretty well understood, even by the laity, so that generally, as far as possible, it is carried out, and with great benefit too. But for this fact thousands would die in their early years who are now, in these days of better intelligence, saved. We are continually urging upon parents, and very properly, the importance of all hygienic points in the management of their families, particularly proper exercise, good air, and suitable food and clothing. The importance of proper food none will question, and it may be affirmed that the mother's milk, if healthy, and cow's milk, where it can be obtained pure, is all that is required. In regard to the various "foods" that are advertised so extensively, I have little experience, having, first, a strong prejudice against all such preparations for infants and children, because I believe nature to be the best chemist in providing her own nutriment, and secondly, because milk has invariably proved itself all sufficient.

Proper clothing is of equal importance, but it is readily understood that all these requisites cannot be carried out by a large proportion of the families, on account of pecuniary inability. Good air, good food, and good clothing are not the gifts of poverty. Fortunately, however, milk is, in most places, abundant and cheap, and a portion of it each day, with even coarse food, will build up many a poor body.

Aside from such judicious management, I know of none that offers to us more promise of success than a persevering course of cod-liver oil. After much careful observation of its effects I can confidently affirm that cases such as we have been considering form a large class, in which the benefits of this *great remedy* are too little understood. Not that its value is underrated in the various and actual *diseases* that are commonly called scrofulous; not that it is neglected when disease has fairly invaded the system; for these all acknowledge its potency for good; but as a *modifier* of the whole organization, *before* disease has invaded it, even while it is yet unsuspected, and during the early months of life, then it is that this remedy is most valuable. As Prof. Geo. B. Wood says of mercury, so I say of this remedy, it "revolutionizes" the whole body, builds up every cell, reorganizes, so to speak, the vital forces, and makes the child apparently another being. Therefore, as a preventive, as a fortifier, I would recall the attention of the profession to it, believing, as I do, that it is too much neglected.

From happy observation, in very many instances, I have found it to so enhance the powers of endurance that disease would not take on so readily fatal forms, or meet with so many complications. With this understanding of the relation of cod liver oil to such constitutions, the natural conclusion is that the sooner its beneficial effects can be realized, the earlier it can be given, the more hopeful we may be of success.

In order to illustrate properly my experience in the matter, I will cite an instance, one of a number that could be given. It is a family by the name of Butler; the father and mother apparently healthy; had a child, the first, that with some evidences of a delicate constitution, has grown to be a boy of nine years. The second child, at eleven months of age, died of marasmus. The third began to decline at four months, after being urged to administer cod-liver oil. This the mother neglected, or rather refused to do. A few months after it was attacked with dysenteric symptoms and died. After the birth of the fourth child the parents were urged still more strongly to administer the remedy early. It was neglected again, and this one died at the age of eleven months. She then promised if another should be born she would administer the remedy. She did so for several months, commencing soon after its birth, and it has now passed three summers, and is healthy. She has now the sixth, thirteen months old, to which was also given the oil faithfully, and which is as promising as the last.

I think the remedy should be administered early ; if possible, long before the first summer, and continued diligently, in such doses as the little stomach will bear. I have not generally discontinued its use upon the occurrence of an ordinary diarrhœa or dysentery, for, with Ringer, I find it often seems to benefit such cases, especially the latter. It is needless to say it should be continued several months, if possible.

To omit the discussion of the operation of cod-liver oil is excusable, as our present knowledge has not enabled us to come to any satisfactory conclusion in other diseases in which its beneficial effects are acknowledged. Certainly, it is a compound of many and valuable constituents. According to De Jongh (U. S. Dispensatory,) these consist of iodine, bromine, chlorine, phosphoric and sulphuric acids, phosphorus, lime, magnesia, soda, iron, glycerine, various biliary principles, a peculiar substance, called gaduin, several other peculiar substances not named, etc. The thousands of gallons that are consumed every year is certainly good proof of the high esteem in which it is held by the profession as well as the public, even though its operation is somewhat mysterious.

I am aware that such a general recommendation of this remedy nearly approaches empyricism. And at this point I wish to call attention to the remarks of Niemeyer, that cod-liver oil is used in scrofulous diseases too indiscriminately ; that it is needless to administer it to any but those who are *lean*, with little or no adipose tissue, etc. This is undoubtedly true in reference to tubercular affections, but I must dissent from it in the cases I have mentioned, especially in infants and young children who have *not* yet reached the tubercular or disease stage.

Many other points bearing upon this subject might be discussed, but I shall take time to offer a very few remarks upon only one, and that is, weaning from the mother. Much might here be said in quoting the opinion of others, but I must be content in stating some practical points which have impressed themselves after many years of observation.

It is evident that a healthy mother, with healthy milk, should nurse her own child; on the other hand, if unhealthy, she should not. But can we always determine accurately this point? It is a fact that a healthy, or, certainly, an apparently healthy mother, may secrete unhealthy milk. Such cases have come under my own observation, and doubtless many others have noticed the same thing.

Dr. Jacobi, of New York City, has recently (*American Journal of Obstetrics*, volume xii, page 541) given some indispensable instructions, which all must appreciate, and which very clearly intimate an endorsement of the points in infant therapeutics, etc., upon which I am speaking.

Bedford, in his "Diseases of Women and Children," gives excellent advice on lactation and weaning, which it would do well for all to consider. He refers to a child suffering with a persistent diarrhœa which apparently had baffled ordinary treatment. He examined the mother's milk and found it to contain colostrum, which should be found in the breasts for only the first few days after delivery, as is well known. He weaned the child and the cure followed spontaneously.

Similar facts I have known in regard to cow's milk. One family had two cows; from one the child was fed and did well. The other cow's milk acted speedily and always as a cathartic, and the mother told me that whenever she desired to administer a cathartic to her child she gave it the milk from this second cow. Both cows were equally healthy. Another family had a cow the milk from which always produced an eruption on the child's skin. After several trials and experiments, the milk was necessarily abandoned. Why may not human milk sometimes present the same anomalies?

The truth is, we may estimate approximately, never with precision; therefore, when a child fails to do well with good medical advice, it is certainly good practice to wean it from the mother, unless the time approaches too near the hot months, and either find a wet nurse, or, better still, generally, raise it on cow's milk. Indeed, I generally feel a sense of relief when any circumstance induces parents to rear their scrofulous or tainted children on cow's milk. This I am willing to repeat with emphasis, having long ago formed the belief that mothers in these modern days are too commonly unfit to nurse their children.

It must be acknowledged that one very serious obstacle exists to the general adoption of this advice. It is the changed, unnatural condition of cow's milk as found in the larger cities; a change that appears to be decided and unmistakable. While this fact is sufficiently known and acknowledged, it is undoubtedly true that physicians in such cities scarcely appreciate it in its full length and breadth.

Milk in the city and country is, indeed, too different fluids; different in nearly all points. The fat is more irritating, because more indigestible; the casein forms in the stomach in larger and denser masses; even the salts are not in such perfect solution, besides being chemically changed by the external forces to which city milk is necessarily subjected. It is often rendered unfit for any stomach, especially that of the tender infant,

In the country the milk is at once and carefully carried to a cool, dark cellar, strained, placed in pans, and left undisturbed, and away from light and heat. It is scarcely changed, chemically or physically, from one milking to the next. It is simply sweet, pure, fresh, untouched, perfect. In the city it is in every respect exactly the reverse, particularly in warm weather; the long agitation in its transportation, the heat, the light, and too often the adulteration with chalk, magnesia, water, or whatever the peculiar "receipt" of different dealers directs, rendering it a pabulum entirely changed from the condition in which nature left it. Hence we might well say: In the city raise an infant on breast milk, in the country on either, all things being equal.

It is easy to perceive why such a discrepancy exists in instructions of writers on the management of infants; one having never known or tasted of pure country milk, the other entirely ignorant of the quality of milk as vended in the cities.

It is equally easy to understand, also, why a removal from the city to the country will often rapidly benefit the sick child, the food, especially the milk, more than the air, in my humble opinion, conducing to this happy result. In the latter place the question of infant dietetics becomes much more easy, and, therefore, the therapeutical one proportionately simplified. Even the milk obtained from a cow kept in the city is often injured while in the udder, by driving her a long distance from pasture. She is almost sure to be fed more or less on slop, which is so easily obtained from neighboring dwellings, besides being kept at night in small, smothering stables.

All this, however, does not invalidate the fact, as I deem it, that pure, fresh cow's milk is only second, if not fully equal, to the material furnished by the mother if diluted with pure water for the first four months, afterward given in its full strength.

What, then, it is asked, is the best thing that can be done with infants in the large cities, who are obliged to remain? The answer may readily suggest itself, if we fully comprehend the causes which produce deterioration of milk. Imitate, as far as possible, the treatment it receives in the country, and particularly among small dairy-men, where only it is to be found in its greatest perfection. Avoid agitation, admixture, heat and light, and certainly all this can be done to a considerable extent. First, the supply for the family should be taken fresh from the cow, at least twice a day, carried by hand. and as short a distance as possible; secondly, this should be done in an absolutely cool and cooling receptacle, and third, it should be kept carefully in a cool cellar, or in a common refrigerator, from which it is to be taken only in small quantities, as required, warmed and given to the child. I am satisfied, from personal observation, that such precautions will repay the little extra trouble involved.

The conclusions which may be drawn from clinical experience, and to which I invite attention, are—

1. That "taints" exist much more frequently in children of families than in the parents.

2. That such taints produce a low grade of vitality.

3. That among these taints or defects scrofula and sexual exhaustion are the most common.

4. That external appearances and the family history do not always point to these constitutional effects.

5. That the unnatural and ready yielding of the body to disease is often the first evidence we have of the existence of taint.

6. That disease in such a body is not what the same disease is in one that is free from such fault.

7. That cod-liver oil is often capable of neutralizing such faults, or in other words, of revolutionizing the vital forces.

8. That to accomplish this the remedy is to be given early in life, and *before evident disease commences.—Med. & Surg. Reporter.*

